# Complex deformation of cartilage micropellets following mechanical stimulation promotes chondrocyte gene expression

**DOI:** 10.1186/s13287-023-03459-5

**Published:** 2023-08-30

**Authors:** Noémie Petitjean, Patrick Canadas, Christian Jorgensen, Pascale Royer, Simon Le Floc’h, Danièle Noël

**Affiliations:** 1 IRMB, University of Montpellier, INSERM, Montpellier, France; 2 LMGC, CNRS, University of Montpellier, Montpellier, France; 3 Clinical Immunology and Osteoarticular Disease Therapeutic Unit, Department of Rheumatology, CHU Montpellier, Montpellier, France; 4 Inserm U1183, IRMB, Hôpital Saint-Eloi, 80 Avenue Augustin Fliche, 34295 Montpellier Cedex 5, France

**Keywords:** Biomechanics, Cartilage micropellet, Mesenchymal stromal cells, Fluidic device, Mechanical stimulation, Differentiation

## Abstract

**Background:**

Articular cartilage (AC)’s main function is to resist to a stressful mechanical environment, and chondrocytes are responding to mechanical stress for the development and homeostasis of this tissue. However, current knowledge on processes involved in response to mechanical stimulation is still limited. These mechanisms are commonly investigated in engineered cartilage models where the chondrocytes are included in an exogeneous biomaterial different from their natural extracellular matrix. The aim of the present study is to better understand the impact of mechanical stimulation on mesenchymal stromal cells (MSCs)-derived chondrocytes generated in their own extracellular matrix.

**Methods:**

A fluidic custom-made device was used for the mechanical stimulation of cartilage micropellets obtained from human MSCs by culture in a chondrogenic medium for 21 days. Six micropellets were positioned into the conical wells of the device chamber and stimulated with different signals of positive pressure (amplitude, frequency and duration). A camera was used to record the sinking of each micropellet into their cone, and micropellet deformation was analyzed using a finite element model. Micropellets were harvested at different time points after stimulation for RT-qPCR and histology analysis.

**Results:**

Moderate micropellet deformation was observed during stimulation with square pressure signals as mean von Mises strains between 6.39 and 14.35% were estimated for amplitudes of 1.75–14 kPa superimposed on a base pressure of 50% of the amplitude. The compression, tension and shear observed during deformation did not alter micropellet microstructure as shown by histological staining. A rapid and transient increase in the expression of chondrocyte markers (*SOX9*, *AGG* and *COL2B*) was measured after a single 30-min stimulation with a square pressure signal of 3.5 kPa amplitude superimposed on a minimum pressure of 1.75 kPa, at 1 Hz. A small change of 1% of cyclical deformations when using a square pressure signal instead of a constant pressure signal induced a fold change of 2 to 3 of chondrogenic gene expression. Moreover, the expression of fibrocartilage (*COL I*) or hypertrophic cartilage (*COL X*, *MMP13* and *ADAMTS5*) was not significantly regulated, except for *COL X*.

**Conclusions:**

Our data demonstrate that the dynamic deformation of cartilage micropellets by fluidic-based compression modulates the expression of chondrocyte genes responsible for the production of a cartilage-like extracellular matrix. This lays the foundations for further investigating the chondrocyte mechanobiology and the cartilage growth under mechanical stimulation.

## Introduction

Articular cartilage (AC) is a highly organized connective tissue, which covers the long bones, ensures smooth movements and facilitates efficient transmission of forces in the joint. These properties are explained by the complex mechanical behavior of AC and primarily depend on its composition and structure [1]. The development and homeostasis of AC depend on the metabolic activity of chondrocytes, which is sensitive to mechanical stimuli. However, the difficulty to reach and maintain in vitro a stable chondrocyte phenotype even under mechanical stimulation highlights the need for a better understanding of chondrocyte mechanobiology using a relevant in vitro model of cartilage growth.

The cartilage micropellet is a relevant and widely used model to study in vitro the growth of AC after differentiation of mesenchymal stromal cells (MSCs) into chondrocytes [2]. This model recapitulates the different stages of cartilage development from MSC condensation to proliferation and differentiation into chondrocytes. Chondrocytes secrete the specific extracellular matrix (ECM) characterized by the production of type II collagen, aggrecan and other proteoglycans. Nevertheless, the cartilage micropellet does not totally reproduce a mature adult AC and evidence for fibrocartilage or hypertrophic cartilage formation has been reported [3–5]. Efforts have been made to limit hypertrophic differentiation by using sequential exposures of different growth factors [6–8]. Still, the composition and organization of the micropellet ECM remain heterogeneous. This is consistent with the few studies that have evaluated the mechanical properties of micropellets and their poor stiffness that is closer to post-natal AC than to adult AC [9–12]. Since biomechanical stimulation is essential for embryonic and post-natal development toward adult native AC, mechanical stimulation is likely the missing parameter in cartilage engineering strategies for improved ECM secretion and organization [13, 14].

The impact of mechanical stimulation on cartilage micropellets has poorly been investigated. Although AC is stimulated by compression, tension and shear deformations in vivo, the techniques used in vitro to stimulate micropellets generate slight mechanical deformations [15]. To our knowledge, only two types of mechanical stimulation have been used: hydrostatic pressures (5 or 10 MPa) and weak electromagnetic fields (2 mT), one or four days after micropellet formation [16–20]. Both types of dynamic stimulation with different intensities, frequencies and durations induced an increased expression of chondrocyte genes (Sox9, aggrecan and type II collagen). Repeated hydrostatic pressures at 0.5 or 1 Hz, for 4 h per day for several days were effective for chondrocyte marker upregulation, while only one ten-minute long electromagnetic stimulation, at 15 Hz, was effective to stimulate gene expression after 1 and 3 weeks. Magneto-mechanical stimulation of MSCs has been applied to enhance osteogenic differentiation but no study reports on chondrogenesis [21]. In any case, the impact of mechanical stimuli that generate greater deformations has not been investigated yet on cartilage micropellets.

In the present study, the objective was to mechanically stimulate cartilage micropellets by using a home-made device that has been previously validated for cell-free biomaterial-based microspheres [22]. First, we evaluated the impact of different signal parameters of biomechanical stimulation on the deformation of micropellets and on the expression of ECM-related chondrocyte genes. Then, we assessed the impact of different types of signal with the selected parameters. Finally, we evaluated the regulation of other cell phenotypes following the mechanical stimulation of micropellets.

## Materials and methods

### MSC isolation and chondrogenic differentiation in micropellets

Human MSCs were isolated from the bone marrow of a single 40-year-old female bone marrow donor, after informed consent and approval by the French Ministry of Research and Innovation and the Personal data Protection ethics Committee (CPP) of Languedoc-Roussillon (Project Arthrocart; approval DC-2010-1185 on 09/21/2010). Briefly, cells were flushed out from the trabecular bone tissues and filtrated on a 70 µm porous membrane (Cell Strainer, Corning, Boulogne-Billancourt) before centrifugation at 300 g for 10 min. Cells were cultured in α-MEM containing 10% fetal calf serum, 2 mM glutamine and 10 µg/mL bFGF. BM-MSCs were characterized by their immunophenotype CD11b^−^/CD19^−^/CD34^−^/CD45^−^/CD73^+^/CD90^+^/CD105^+^ and trilineage differentiation potential as previously described [23]. The cells were expanded and used at passage 5 after medium changes twice a week, as described [24]. Chondrogenic differentiation was induced using the 3D micropellet model by centrifuging 2.5 × 10^5^ MSCs in 15 ml conical tubes at 300 g for 5 min. Chondro-inductive medium (DMEM high glucose, 0.1 µM dexamethasone, 1 mM sodium pyruvate, 170 µM ascorbic-2-phosphate acid, 1% insulin/transferrin/selenic acid and 0.35 mM proline) supplemented with 10 ng/mL transforming growth factor β3 (TGFβ3) (Bio-Techne, Lille) was changed every 3 days for 21 days as described [25].

### Mechanical stimulations

#### Mechanical loading device

A home-made device, previously validated [22], was used to stimulate the micropellets. Briefly, this is a fluidic system, which consists of a 3D-printed tank with 6 conical wells for the concomitant stimulation of 6 micropellets. Positive pressure was applied at the top of the micropellets, while atmospheric pressure was maintained at the bottom. The difference in pressure, measured with a pressure sensor (ADP5121, Panasonic, Farnell, Limonest, France), caused the micropellets to sink into their cones and be deformed. A camera (Mako, Allied Vision, Stemmer Imaging, Suresnes, France) placed in front of the tank automatically moved and took pictures of each micropellet. The medium contained in the whole circuit (16 mL) was activated by 2 peristaltic pumps (15KS series, Boxer, Flow Technique, Entzheim, France). The device was held at 37 °C and 5% CO_2_ in a humidified cell culture incubator.

#### Mechanical stimulation with different signal parameters

Cartilage micropellets were mechanically stimulated once at day 21 of differentiation. At this time, the strength of the micropellets was sufficient to withstand the mechanical stimulation [10]. The stimulation was performed in the tank that contains 6 micropellets at the same time. Micropellets were subjected to cyclic pressure in a square waveform with different amplitudes (1.75, 3.5, 7 and 14 kPa), frequencies (0.25, 0.5, 1 and 2 Hz) and durations (15, 30 and 60 min). Each parameter was tested independently keeping a middle value for the others, as indicated in Table 1. The square signal was overlaid with a minimum pressure (*P*_m_) of 50% of the signal amplitude in order to keep micropellets in their cones. Stimulations were conducted with alternate cycles of 180 s of pressure and 23 s of rest at *P*_m_ for a total of 30 min of stimulation (10 cycles). A free-swelling control consisted of a group of micropellets left in the device without stimulation. Thereafter, micropellets were returned to their tubes for 24 h before recovery for analysis.

**Table 1:** Parameters of square pressure signal tested in the fluidic device

Duration (*A* = 7 kPa, *F* = 1 Hz) (min)	Frequency (*A* = 7 kPa, *T* = 30 min) (Hz)	Amplitude (*F* = 1 Hz, *T* = 30 min) (kPa)
15	0.25	1.75
30	0.5	3.5
60	1	7
	2	1

Each parameter (duration, frequency, amplitude) was tested while keeping the mean parameter for the others as indicated in the first raw

#### Mechanical stimulation with different times of analysis

Cartilage micropellets (groups of 6) were mechanically stimulated once at day 21 of differentiation. They were subjected to cyclical pressure in a square waveform with 3.5 kPa amplitude, 1 Hz frequency and 30 min duration. Micropellets were recovered at 0, 3, 6, 15 or 24 h after stimulation.

#### Mechanical stimulation with different shapes of pressure signal

Cartilage micropellets (groups of 6) were mechanically stimulated once at day 21 of differentiation. They were subjected to cyclical pressure in a square, sinusoidal or constant waveform for 30 min. Dynamic signals had an amplitude of 3.5 kPa and a frequency of 1 Hz. The constant signal had an amplitude of 3.5 kPa corresponding to the mean pressure of the dynamic signals. Micropellets were recovered at 3 h after stimulation.

### RT-qPCR

Each group of 6 micropellets was washed in phosphate buffered saline (PBS) and stored at − 80 °C before RT-qPCR analysis. Total RNA was extracted using the RNeasy microkit (Qiagen, Courtaboeuf) after mechanical dissociation using Ultra-Turrax homogenizer. Total RNA (0.2 µg) was then reverse transcribed using the M-MLV reverse transcriptase (ThermoFisher Scientific, Villebon-sur-Yvette). Primers for chondrocyte markers were designed using the Primer3 software (Table 2) and purchased from MWG (Eurofins Genomics, Courtaboeuf). PCR reactions were carried out using 10 ng of cDNA, 5 µmol/L of each primer, and 5 µL 2X SYBR Green PCR Master Mix (Roche, Meylan). The following cycling conditions were used: 95 °C for 5 min; then 40 cycles at 95 °C for 15 s; 64 °C for 10 s, and 72 °C for 20 s in a Viia7 Real-Time PCR System (Life Technologies, Courtaboeuf). Gene expression was analyzed using the comparative Ct method (2^−ΔΔCt^) after normalization to the housekeeping gene RSP9 and comparison with the non-stimulated control group.

**Table 2:** Primer sequences used for RT-qPCR experiments

Gene symbol	Gene name	Sequence forward	Sequence reverse
ADAMTS5	A Disintegrin and Metalloproteinase with Thrombospondin motifs	CTCCACGCAGCCTT-CACTGT	TGGGTGGCATCGTA-GGTCTG
AGG	Aggrecan	TCGAGGACAGCGAGGCC	TCGAGGGTGTAGCGT-GTAGAGA
AP	Alkaline phosphatase	CCACGTCTTCACAT-TTGGTG	GCAGTGAAGGGCTT-CTTGTC
COL I	Collagen type I	CCTGGATGCCATCA-AAGTCT	CGCCATACTCGAAC-TGGAAT
COL IIB	Collagen type IIB	CAGACGCTGGTGCTGCT	TCCTGGTTGCCGGACAT
COL X	Collagen type X	TGCTGCCACAAATA-CCCTTT	GTGGACCAGGAGTA-CCTTGC
LINK	Link protein	TTCCACAAGCACAA-ACTTTACACAT	GTGAAACTGAGTTT-TGTATAACCTCTCAGT
MMP13	Matrix Metallopeptidase 13	TAAGGAGCATGGCG-ACTTCT	GTCTGGCGTTTTTG-GATGTT
RSP9	Ribosomal small protein 9	ATGAAGGACGGGAT-GTTCAC	GATTACATCCTGGG-CCTGAA
RUNX2	Runt-related transcription factor 2	CGGAATGCCTCTGC-TGTTAT	TTCCCGAGGTCCAT-CTACTG
SOX9	Sex Determining Region Y-Box 9	AGGTGCTCAAAGGC-TACGAC	GTAATCCGGGTGGT-CCTTCT
SPARC	Secreted protein, Acidic, Cysteine-Rich	AAAGCACAAGGCAG-AAAGGA	GGTGGGCTTGATGA-CTCTGT

### Histological analysis

Micropellets were fixed in 3.7% formaldehyde for 1 h and processed for routine histology. Deparaffinized micropellet sections (3 µm) were stained with Safranin O/Fast Green.

### Biomechanical analysis

#### Finite element model

An axisymmetric finite element model has previously been created with the software LMGC90 [22]. The numerical model simulates the sinking of a micropellet, represented by half a disk meshed by the software *GMSH* with 1760 triangles and employing a neo-Hookean hyperelastic law, in the cone [26]. The cone is simulated by rigid wells. A friction-less contact law is used between the micropellet and the cone.

#### Deformation of micropellets caused by loading

Twenty images of each micropellet, equivalent to ten images per period of pressure signal, were recorded in the middle of each cycle of stimulation. The oscillations of micropellets following the dynamics of pressure signal were analyzed by determining the resulting displacements of the top and bottom ends of micropellets through image analysis using a *python* code with *OpenCV* library (*threshold* function). The average sinking of micropellets at the beginning (first stimulation cycle) and the end (last stimulation cycle) of the 30 min stimulation was determined by calculating the average displacement of the bottom of micropellets. All displacements were normalized to micropellet diameter, calculated from the area of the micropellet before applying pressure. As described in a previous study, the displacement fields only depend on the ratio of the pressure to the Young’s modulus and on the Poisson’s ratio [22]. The mean value of the Poisson’s ratio of 0.45 was used, as identified for 21-day micropellets. The displacement fields were then estimated, thanks to the finite element model, at a particular value of the pressure ratio to the Young’s modulus. This value was identified so that the displacement of the bottom of the simulated microsphere corresponded to the mean one measured experimentally on micropellets. The strains of the micropellets in radial, circumferential and longitudinal directions, von Mises strains and their change of volume were recorded using the finite element model.

### Statistical analysis

GraphPad Prism 9 software (San Diego, USA) was used to create images and perform the statistical analyses. Since data did not pass the D'Agostino–Pearson omnibus normality test, the nonparametric one sample Wilcoxson signed rank test was used to compare the gene expression of the 1-day and 21-day micropellets and the gene expression of the stimulated and non-stimulated samples. When all groups passed the D'Agostino–Pearson omnibus normality test, the multiple comparison test between displacement measures was performed with the one-way ANOVA test and comparison between two measurements of a same group with the paired t test. When groups did not pass the D'Agostino–Pearson omnibus normality test, the multiple comparison test between displacement measures was performed using the Kruskal–Wallis test. Comparison between two measurements of a same group was done using the nonparametric Wilcoxson signed rank test.

## Results

### Mechanical stimulations induced micropellet deformation

**Fig. 1:**
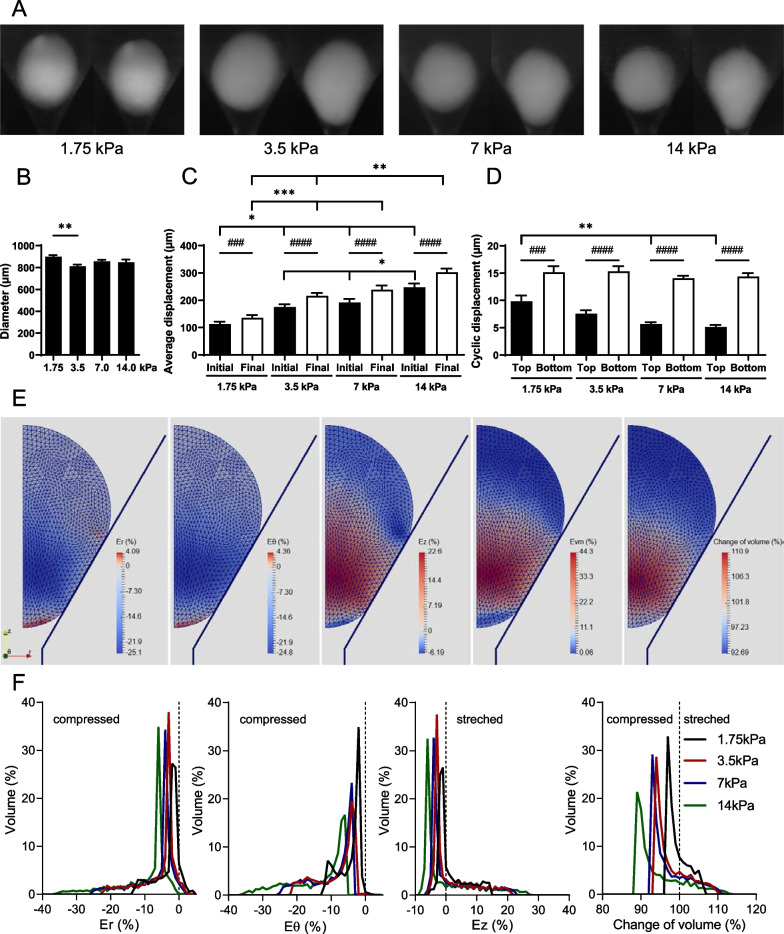
Deformation of cartilage micropellets induced by mechanical stimulations with square pressure signals of different amplitudes. **A** Sinking of micropellets in their cones observed with a 2.65 µm resolution camera. **B** Average diameter of micropellets studied in each group (*n* = 11–20 micropellets). **C** Average sinking of micropellets in their cones at the beginning (Initial) and end (Final) of 30 min of stimulation depending on the pressure amplitude (*n* = 11–20 micropellets). **D** Average amplitude of cyclic oscillations of the micropellet top and bottom ends depending on pressure amplitude (*n* = 11–20 micropellets). **E** Localization of radial (Er), circumferential (Eθ) and longitudinal (Ez) strains, the von Mises strains (Evm) and the volume change of micropellets during stimulation with the 7 kPa pressure amplitude, from left to right respectively, shown by a finite element model. (F) Distribution in volume of Er, Eθ, Ez and volume change in micropellets depending on pressure amplitudes and estimated by the finite element model. Statistical analysis used Kruskal–Wallis test (*: *p* < 0.05, **: *p* < 0.01, ***: *p* < 0.001) or Wilcoxon matched-pairs signed rank test (###: *p* < 0.001, ####: *p* < 0.0001)

A systematic analysis of images was made to determine the range of micropellet deformations for the different amplitudes of stimulation. A simple observation of the images taken with the camera confirmed that micropellets were deformed whatever the pressure amplitude (Fig. 1A). Before applying pressure, micropellet diameters were recorded in all groups (Fig. 1B). The sinking of micropellets in their cones during stimulation can be described as an average sinking superimposed by oscillations, which are 22-fold smaller (Fig. 1C, D). The sinking of micropellets, as shown by their displacement, was significantly increased between the beginning (Initial) and the end (Final) of stimulations in all groups (Fig. 1C). Moreover, the sinking of the “1.75 kPa” micropellet group was significantly lower than the others. The sinking of the “3.5 kPa” and “7 kPa” groups is similar but significantly lower than the “14 kPa” group, at the beginning of stimulations. The oscillations of micropellet bottom ends were significantly higher than the oscillations of micropellet top ends (Fig. 1D). The oscillations of micropellet top ends were significantly higher for the “1.75 kPa” groups compared to the “7 kPa” and “14 kPa” groups (Fig. 1D).

Local strains on micropellets were then estimated using the finite element model. During stimulations, the micropellet was compressed in the radial (Er) and circumferential (E*θ*) directions by the shape of the cone, except for a thin layer at the bottom of the micropellet and the small area that is brought into contact with the cone (Fig. 1E). In the longitudinal direction (Ey), the upper part and the thin layer at the bottom were compressed, whereas the rest of the micropellet was stretched. The spread of the von Mises strains shows that the middle part of the micropellet, from its center to the contact zone with the cone, underwent higher deformations than the upper part and the thin layer at the bottom. These deformations created an increased volume of the middle part, while the upper part and the thin layer at the bottom decreased in volume.

The distribution of strains in each direction, in terms of volume, and the change of volume as a function of micropellet sinking were analyzed by tracking the strain and volume of the elements in the numerical model, using the average sinking (Fig. 1F). As expected, the strain amplitudes increase with the increase in the applied pressure as shown by the increase in compressions in all directions and the volume change. Maximal compression exceeded 30% in radial and circumferential directions for the “14 kPa” group while remaining under 25% for the “3.5 kPa” and “7 kPa” groups and under 15% for the “1.75 kPa” group. By integrating the distribution curves, we evaluated that 15.24%, 0.62% and 29.36% of the volume of the “14 kPa” group were deformed by less than 5% in the radial, circumferential and longitudinal directions, respectively, compared to at least 62.14%, 32.84% and 74.46% for the other groups. For the “3.5 kPa,” “7 kPa” and “14 kPa” groups, strains between 10 and 20% applied to 16.69 ± 0.96%, 27.20 ± 2.49% and 13.39 ± 1.38% of micropellet volume in the radial, circumferential and longitudinal directions, respectively. For the “1.75 kPa” group, this interval represented only 8.79%, 12.54% and 8.67%, respectively. In the different groups, 35.51 ± 1.48% of the micropellets were stretched in the longitudinal direction. The mean of von Mises strain was 6.39%, 10.26%, 11.29% and 14.35% for the “1.75 kPa,” “3.5 kPa,” “7 kPa” and “14 kPa” groups, respectively. Finally, the average volume of micropellets decreased from 99.46 to 94.30% of the initial volume with increasing amplitudes of stimuli. The small oscillations of displacement that were measured in case of cyclic pressure corresponded to much smaller mean of von Mises strain, less than 1%, for all 4 groups. Such small oscillatory strains may have a great influence on gene expression.

### Mechanical stimulations increased the expression of chondrocyte markers

**Fig. 2:**
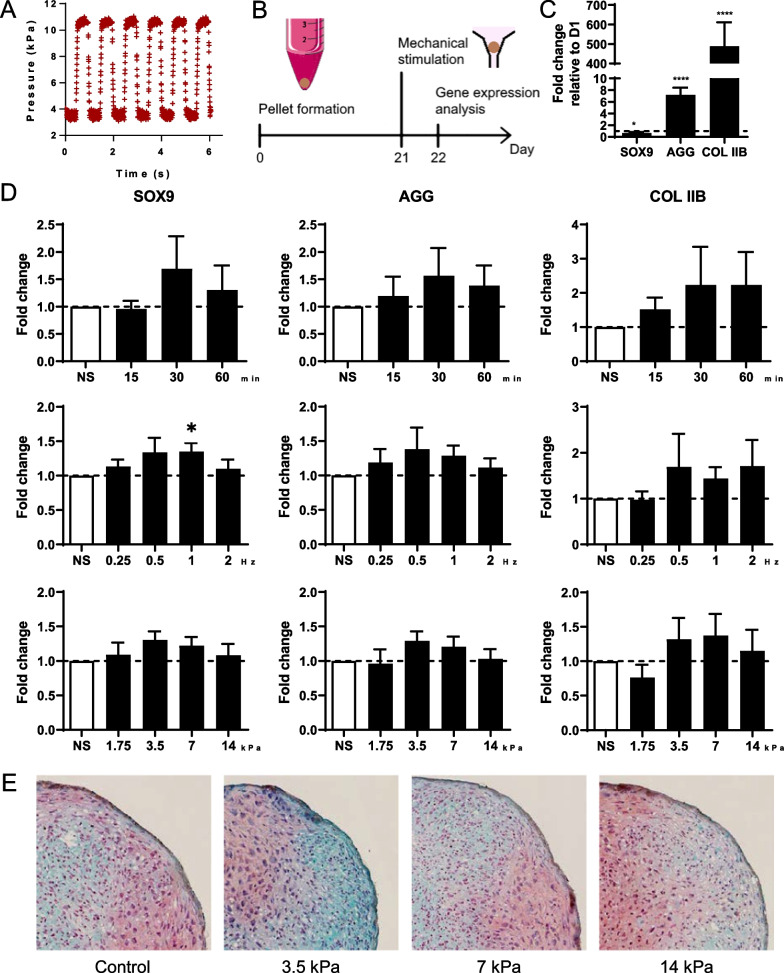
Mechanical stimulations of mesenchymal stromal cells-derived cartilage micropellets with square pressure signals. **A** Representative square-wave pressure signal generated by the device. **B** Experimental design to study the impact of mechanical stimulations with different pressure signal parameters on gene expression in micropellets. **C** Fold change expression of *SOX9*, aggrecan (*AGG*) and type IIB collagen (*COL IIB*) transcripts in micropellets after 21 days of differentiation compared to day 1 (*n* = 13 groups of 6 micropellets). **D** Fold change expression of *SOX9, AGG* and *COL IIB* in micropellets after stimulations with different durations (upper panel), frequencies (middle panel) and amplitudes (lower panel) compared to non-stimulated control (NS), at day 21 (*n* = 4–9 groups of 6 micropellets). **E** Histological staining of proteoglycans by Safranin O and fast green counterstaining in 21-day micropellets depending on pressure amplitude. Statistical analysis used Wilcoxson signed rank test, and results are expressed as *: *p* < 0.05, ****: *p* < 0.0001

Mechanical stimulation with different square-wave pressure signals was performed on cartilage micropellets on day 21 (Fig. 2A, B). Chondrocyte differentiation of MSCs cultured in micropellets was confirmed by the upregulation of the two main articular cartilage ECM markers (aggrecan (*AGG)* and type IIB collagen (*COLIIB*)), while *SOX9* expression was downregulated at day 21 (Fig. 2C). The impact of different durations, frequencies and amplitudes was tested on the expression of the three chondrocyte genes (Fig. 2D). Although the results were not significant, a duration of 30 or 60 min of mechanical stimulations seemed to increase the expression of the three genes. Stimulations at a frequency of 0.5 or 1 Hz showed the highest upregulation of *SOX9* and *AGG*. The upregulation of *SOX9* was significant for the 1 Hz condition. Finally, a similar trend to increased expression of the three genes was observed at the amplitudes of 3.5 and 7 kPa.

As internal strains of micropellets can exceed 20% in compression or tension depending on their direction when stimulated with an amplitude of 3.5, 7 or 14 kPa, the structural integrity of micropellets was questioned by histological analysis. A peripheral zone with elongated cells and the core zone with characteristic chondrocytes in lacunae were observed (Fig. 2E). Staining with Safranin O/Fast green confirmed that proteoglycans were produced in the micropellets during the 21 days of culture prior to mechanical stimulation as shown by the red staining of the ECM. No obvious structural alteration was noticed in stimulated micropellets compared to the control ones whatever the pressure amplitudes tested. Therefore, subsequent stimulations of micropellets were performed using the following parameters: amplitude of 3.5 kPa and frequency of 1 Hz for 30 min.

### Chondrocyte markers are rapidly upregulated after mechanical stimulations

**Fig. 3:**
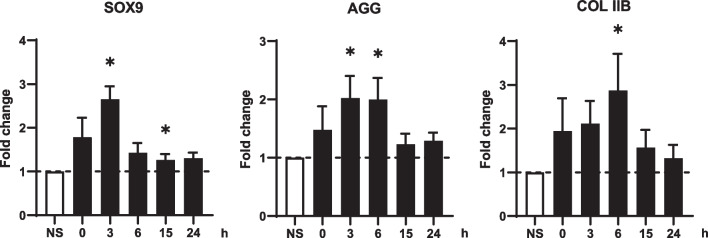
Kinetics of chondrocyte marker expression after mechanical stimulation with a square pressure signal. Micropellets of 21 days were stimulated by a square-wave pressure with an amplitude of 3.5 kPa and a frequency of 1 Hz for 30 min. Expression of *SOX9*, *AGG* and *COL IIB* was evaluated 0, 3, 6, 15 and 24 h after mechanical stimulations and compared to non-stimulated control (NS) (*n* = 7–9 groups of 6 micropellets). Statistical analysis used Wilcoxson signed rank test, and results are expressed as *: *p* < 0.05

Since the increase in gene expression was not significant in previous experiments, we performed an expression kinetics of chondrocyte markers at early time points. The expression of *SOX9*, *AGG*, and *COL IIB* was assessed at 0, 3, 6, 15 and 24 h after mechanical stimulation with a square waveform and parameters determined previously. Upregulation of the expression of all three genes was observed as soon as the stimulation is completed (Fig. 3). Maximal expression of *SOX9* was observed after 3 h, even though *SOX9* expression was still significantly increased after 15 h. The maximal expression peaks for *AGG* and *COL IIB* were observed after 3 and 6 h. These results indicated that mechanical stimulations induced an early gene response and highlighted the 3 h time point as optimal for chondrocyte gene upregulation (by a two–three fold factor). The results also provided evidence that a single 30 min session of mechanical stimulations is sufficient to significantly upregulate chondrocyte genes that are responsible for the production of cartilage ECM.

### Only dynamic signals can upregulate chondrocyte marker expression

**Fig. 4:**
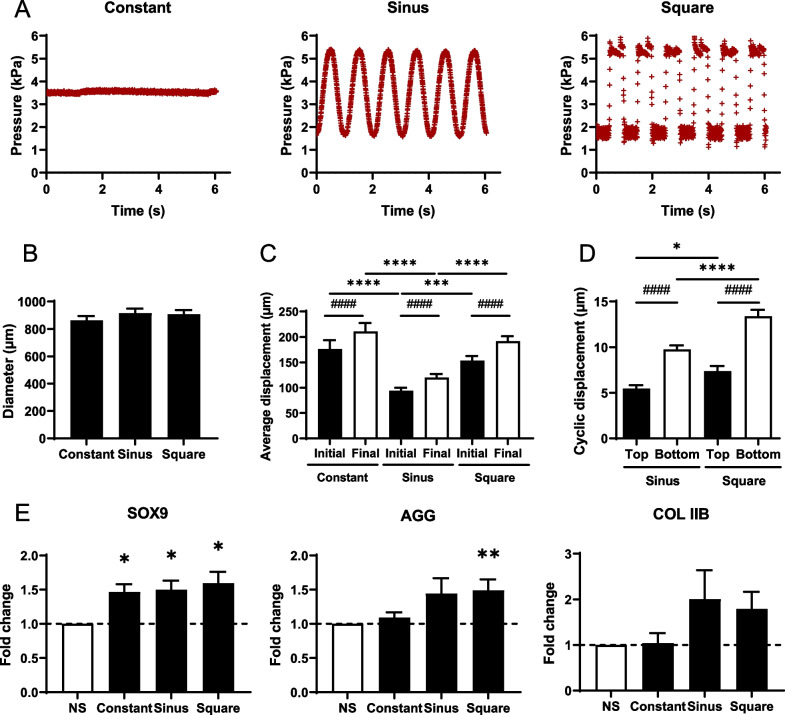
Mechanical stimulations of mesenchymal stromal cells-derived cartilage micropellets with different shapes of pressure signal. **A** Representative constant pressure signal with an amplitude of 3.5 kPa, square and sinusoidal pressure signals with an amplitude of 3.5 kPa and a frequency of 1 Hz that stimulated 21-day micropellets for 30 min. **B** Average diameter of micropellets studied in each group (*n* = 15 micropellets). **C** Average sinking of micropellets in their cones at the beginning (Initial) and end (Final) of 30 min stimulations depending on the shape of the pressure signal (*n* = 15 micropellets). **D** Average amplitude of the cyclic oscillations of the micropellet top and bottom ends depending on the shape of the pressure signal (*n* = 15 micropellets). **E** Fold change expression of *SOX9, AGG* and *COL IIB* in micropellets 3 h after stimulations with different pressure signals compared to non-stimulated control (NS) (*n* = 7–9 groups of 6 micropellets). Statistical analysis used one-way ANOVA test (*: *p* < 0.05, **: *p* < 0.01, ***: *p* < 0.001) or paired *t* test (###: *p* < 0.001, ####: *p* < 0.0001) for **B**, **C** and **D**. Statistical analysis used Wilcoxson signed rank test for **E**, and results are expressed as *: *p* < 0.05, **: *p* < 0.01

Besides generating square signals, we previously reported that constant and cyclical pressure signals can be reliably generated by our device [22]. We therefore evaluated the impact of different types of stimulation signals on chondrocyte gene expression on 21 day micropellets (Fig. 4A). The mean amplitude of the constant signal was 3.76 ± 0.39 kPa. The minimum pressure of the sinusoidal and square signals was 1.55 ± 0.57 kPa and 1.75 ± 0.01 kPa, respectively, and their amplitude was 3.43 ± 0.14 kPa and 3.53 ± 0.03 kPa. Thus, the average pressure applied on micropellets with the sinusoidal and square signals was 3.27 ± 0.63 kPa and 3.52 ± 0.02 kPa, respectively. The frequency of these signals was 1.05 Hz and 1 Hz, respectively. The analysis of the sinking and oscillations of micropellets in their cones for the three signals showed that the average micropellet diameter was not significantly different between groups (Fig. 4B). For each signal, the sinking or displacement of micropellet bottom ends significantly increased during stimulations (Fig. 4C). The sinking of micropellets was significantly higher with the constant and square pressure signals than with the sinusoidal one. We also measured the oscillations of micropellet top and bottom ends for the two dynamic pressure signals (Fig. 4D). As observed previously, the displacement of micropellet bottom ends was significantly higher than that of top ends. Moreover, the displacement of micropellets was significantly higher with the square signal than with the sinusoidal one. Finally, the expression of *SOX9*, *AGG* and *COLIIB* was compared between the different signals, three hours after stimulations (Fig. 4E). *SOX9* was significantly upregulated for the three pressure signals, and the expression of *AGG* and *COLIIB* was enhanced using the sinusoidal and square signals even though the upregulation was significant only for *AGG* using the square signal. Therefore, only the dynamic signals (sinusoidal and square) can upregulate all chondrocyte genes, even though the induced oscillatory strains were very small (< 1%) compared to the constant strains induced by a constant pressure signal (> 10%).

### Articular chondrocyte phenotype is enhanced by mechanical stimulations

**Fig. 5:**
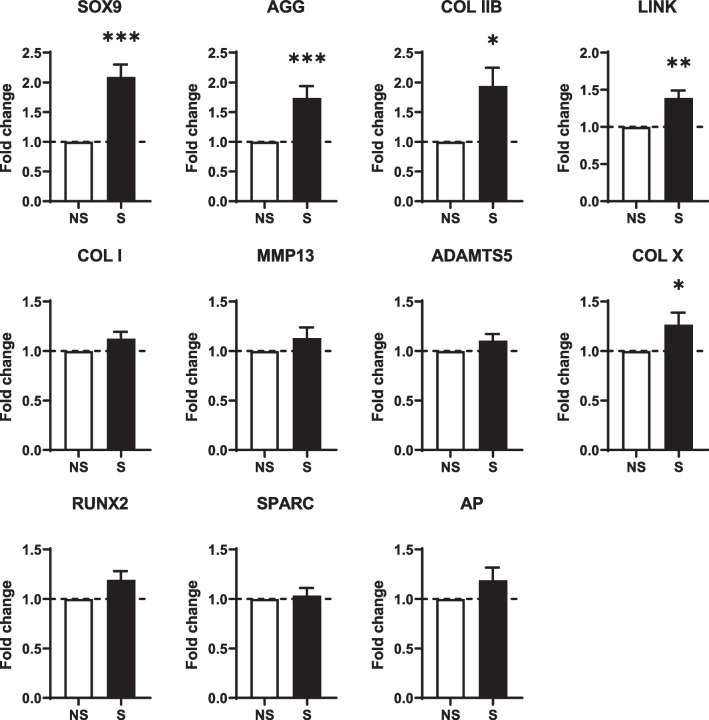
Mechanical stimulations of cartilage micropellets with a square pressure signal specifically upregulated the expression of articular chondrocyte markers. Micropellets of 21 days were stimulated by a square-wave pressure with an amplitude of 3.5 kPa and a frequency of 1 Hz for 30 min. Fold change expression of genes for articular chondrocytes (*SOX9*, *AGG*, *COL IIB* and *LINK*), hypertrophic chondrocytes (*COL X, MMP13*, *ADAMTS5*), fibrochondrocytes (*COL I*) and osteoblasts (*RUNX2*, *SPARC* and *AP*) were analyzed 3 h after stimulations and compared to non-stimulated control (NS) (*n* = 15 groups of 6 micropellets)

Finally, we analyzed in more details the expression of genes specific for mature cartilage, hypertrophic cartilage or bone for micropellets stimulated 30 min by a square signal of pressure of 3.5 kPa amplitude and 1 Hz frequency. The markers of mature chondrocytes (*SOX9, AGG*, *COL IIB* and *LINK*) were all significantly upregulated (Fig. 5). The expression of markers for fibrocartilage (*COLI*) or hypertrophic cartilage (*COL X*, *MMP13* and *ADAMTS5*) was not significantly regulated, except for *COL X*. Similarly, the markers for bone (*RUNX2*, *SPARC* and *AP*) were not modulated by mechanical stimulations. These results indicated that the parameters chosen for mechanical stimulations specifically upregulated the genes of mature articular cartilage without inducing fibrocartilage, hypertrophic cartilage or bone.

## Discussion

We previously validated the interest of a new device dedicated to the mechanical stimulation and characterization of microspheres, in the range of 900–1500 µm [22]. Here, we used the device to explore the response of human MSC-derived cartilage micropellets to different mechanical loading regimes. This is the first study reporting mechanical stimulation and deformation of cartilage micropellets by fluidics as compared to the application of hydrostatic pressure or electromagnetic field [19, 20, 27–29]. This relies on a home-made fluidic-based device that allows reproducible mechanical stimulation to millimeter-sized spheres with imperfect round shape.

Analysis of micropellet displacement during cyclic pressure-driven mechanical stimulations showed an average sinking of micropellets one to two orders of magnitude higher (100–200 µm) than their oscillation (5–15 µm), contrary to the deformations of scaffold-based cartilage constructs from MSCs or chondrocytes observed in compression- or shear-based studies [30–33]. The average sinking of micropellets corresponds to a creep behavior as already noticed by our previous study [22]. As the mean pressure was maintained, the micropellet deformed itself because of its poro–visco–elastic behavior. This creep of micropellets is a well-known mechanical behavior of articular cartilage in both native and engineered tissues [1]. It has already been observed during force-controlled dynamic mechanical stimulations of a cartilage scaffold made from hydrogel embedded-MSCs [34]. Although the average sinking of micropellets was higher with the highest pressure amplitudes, the oscillation amplitude of micropellets tended to decrease, showing a hyperelastic nonlinear behavior, as already reported by our group [10]. In the present study, the measure methods and numerical models can help differentiating cyclic responses from creep responses and thus determine if creep or cyclic responses correspond to higher gene expression.

In addition, using the fluidic-based device, the micropellets are constrained in all directions except around the bottom part of the micropellets in the longitudinal direction, while other studies used constraints applied in one or two directions. Thus, we measured an average global deformation of micropellets with von Mises strains from 6.39 to 14.35% depending on the pressure. The interest of using von Mises strains relies on the fact that transverse and shear strains occur even if a single direction of solicitation is used. Our results remain difficult to compare with the data from literature because (a) the solicitation of a sphere implies (except for hydrostatic pressures) complex non-homogeneous maps of strains that must be locally and globally interpreted through von Mises strain, for example; (b) other studies do not report such von Mises strains that take into account the strains that always occur transversely to the main solicitation direction and thus only report the deformation along the direction of solicitation. In our study, an optimum pressure of 3.5 kPa was obtained, which corresponds to approximately 10% of von Mises strains. This value appears in line with some scaffold-based studies [31–39]. Indeed, the amplitude of deformations in the upper part was similar to that applied on MSC-based cartilage constructs [31, 32, 34]. However, complex and higher deformations occurred in the lower central part of micropellets with maximal deformations exceeding 15% in all directions for the 3.5, 7 and 14 kPa amplitudes, which is in line with other studies. Up to 50% of deformations in one direction or 30% in compression combined with 50% in shear resulted in increased chondrocyte gene expression and matrix accumulation [33, 37, 38]. Of importance, the combination of compression and shear stress, as observed in our study, synergically increased the production of markers of articular cartilage ECM but not those of fibrocartilage or hypertrophic cartilage ECM [33, 38]. Although mechanical stimulations in tension mainly promote osteogenesis, tissue expansion in the transverse direction of compression is a natural deformation resulting from the Poisson effect that is also observed in our settings [15, 35, 36, 39]. Based on the measured displacements and the geometry of wells, the finite element model showed an increased volume of the lower part of micropellets related to a decreased volume of the upper part during mechanical stimulations. This suggests that the pressure generated a fluid flow from the upper part to the lower part, which could promote nutrient exchange in the micropellet core and lead to a more homogeneous cartilage-like tissue over time. Nevertheless, a study showed that forced perfusion could favor hypertrophic cartilage phenotype [40]. Mapping of fluid flow thanks to a model will enhance the comprehension of the development of the micropellet cartilage model.

We conducted a parametric study to evaluate the impact of different stimulation parameters on the expression of chondrocyte markers. Our results are in line with the literature. Although at least one hour of stimulation was generally applied for MSC-based scaffolds, upregulation of *SOX9*, *AGG* and *COL IIB* was already observed after 15 min or 30 min of stimulations [18, 33, 41, 42]. Expression of these genes was higher at the frequency of 0.5 Hz and 1 Hz, while the 1 Hz frequency is the most used since closer to the walking frequency [18, 32, 33, 42, 43]. Frequencies above 1 Hz are rarely used, except for microwave techniques like ultrasounds or electromagnetic fields. Only one report showed higher expression of *AGG* by chondrocytes in agarose gel after stimulation at 3 Hz [44]. Finally, the pressure level used in hydrostatic pressure-based stimulations is around one thousand times higher than the level used in our study (1.75–14 kPa), which is comparable to stress applied in unconfined compression stimulations [16, 18, 20, 27, 34]. The kinetics of gene expression confirmed the early and transient response of chondrocytes to mechanical stimulations with the half-lives of *AGG* and *COL IIB* mRNAs ranging from 7 to 10 h and even lower for *SOX9* mRNA [38, 45]. Importantly, we found that the genes specific for articular chondrocytes were upregulated by mechanical stimulations, while genes specific for fibrocartilage, bone or matrix degradation were not modulated for a rather low pressure of 3.5 kPa (mean value of von Mises strains of 10.26%). These results are of interest since a recent study reported the upregulation of cartilage damage and catabolic signaling after hyper-physiological mechanical stimulations [46]. Therefore, the present parametric study allowed to define the regime of fluidic stimulation (3.5 kPa, 1 Hz, 30 min) for moderate and nondestructive deformation of cartilage micropellets leading to the upregulation of chondrocyte genes.

Dynamic mechanical stimulations of cartilage constructs are usually used since static stimulation has been shown to be less effective for chondrogenesis [34, 47]. Most of the studies used a sinusoidal signal [16, 38, 43, 48–50] and rarely, a square signal [51, 52]. The solicitation rates and the mechanical energy provided by a sinusoidal wave, a square wave or a constant signal are different and might therefore result in different tissue and cell responses. We therefore compared the three types of signal and reported that the average sinking of micropellets was similar to the constant and the square signals and higher than with the sinusoidal signal, while the same average pressure was applied. Regarding the cell response, the dynamic signals were more effective than the static signal in increasing chondrocyte gene expression. As the amplitude of average sinking of micropellets was similar for the square wave and the constant signals, only the cyclic response to the square wave signal could explain the difference of gene expression between constant and square-wave solicitations. Interestingly, only a very small difference of less than 1% of oscillatory deformations between the two mechanical stimulations (constant versus square) induced a high modulation of gene expression. Pressure-driven (and/or force-driven) setups may be more efficient to upregulate gene expression at very low oscillatory strain values than most setups that use displacement (and/or strain) to drive mechanical stimulations. We also observed a significant increase in *AGG* expression only when using the square signal compared to sinusoidal signal. As both the mechanical energy and rate of stimulations induced by square and sinusoidal signals were not exactly the same, we could not conclude that square signals are more effective because they induced higher average strains or cyclic strains. A complementary study, using similar average displacements within the cone for square and sinusoidal signals, could answer such question.

A possible limitation to the present study is the use of a single donor of BM-MSCs. It allowed to reduce the heterogeneity between experiments but the impact of mechanical stimulations on several biological replicates would need to be analyzed in futures studies. A single period of mechanical stimulation was used to assess the feasibility to mechanically stimulate the growth of cartilage micropellets. We observed the upregulation of genes responsible for cartilage-like ECM production, but the stimulation period was too short to assess the production of ECM components at the protein level. Thus, the current protocol can be used to assess mechanotransduction pathways and can be optimized to evaluate the impact of multiple stimulations per day or repeated stimulations over time. Future studies will assess ECM protein secretion and organization, as well as the mechanical properties of cartilage micropellets.

## Conclusion

The present study provides the proof of concept that a custom-made fluidic-based device can be used to mechanically stimulate human MSC-derived cartilage micropellets by deformation. The optimal parameters of pressure signal were defined to get a significant upregulation of chondrocyte markers without inducing genes responsible for matrix degradation or calcification. The study would benefit from a complementary analysis of mechanotransduction pathways involved in cartilage regulation. The device paves the way to studies for better understanding the impact of mechanical stimulation on in vitro chondrogenesis of BM-MSCs in simple models, such micropellets or biomaterial-based scaffolds, in short or long term. The device could be optimized to a more user-friendly and accessible version to be used for enhancing cartilage formation or evaluating the interest of various cartilage tissue engineering approaches in an environment biomechanically closer to the clinical situation.

## Data Availability

All data generated and/or analyzed during this study are available from the corresponding author upon reasonable request.

## Abbreviations

AC: Articular cartilage
AGG: Aggrecan
ADAMTS: A Disintegrin and Metalloproteinase with Thrombospondin Motive
AP: Alkaline phosphatase
COL: Collagen
ECM: Extracellular matrix
MMP: Matrix metalloproteinase
MSCs: Mesenchymal stromal cells
Pm: Minimum pressure
RT-qPCR: Reverse transcriptase-quantitative polymerase chain reaction
RUNX2: Runt-related transcription factor 2
SOX9: Sex determining region Y-box 9
SPARC: Osteonectin
